# Community engagement for malaria elimination in the Greater Mekong Sub-region: a qualitative study among malaria researchers and policymakers

**DOI:** 10.1186/s12936-022-04069-x

**Published:** 2022-02-14

**Authors:** Nils Kaehler, Bipin Adhikari, Phaik Yeong Cheah, Lorenz von Seidlein, Nicholas P. J. Day, Arjen M. Dondorp, Christopher Pell

**Affiliations:** 1 Mahidol-Oxford Tropical Medicine Research Unit, Faculty of Tropical Medicine, Mahidol University, Bangkok, Thailand; 2 Centre for Tropical Medicine and Global Health, Nuffield Department of Medicine, University of Oxford, Oxford, UK; 3 The Ethox Centre, Nuffield Department of Population Health, University of Oxford, Old Road Campus, Oxford, UK; 4 Amsterdam Institute for Global Health and Development (AIGHD), Amsterdam, The Netherlands; 5 Centre for Social Science and Global Health, University of Amsterdam, Amsterdam, The Netherlands

**Keywords:** Policymakers, Community engagement, Malaria, Research, Intervention, Participation, Population coverage

## Abstract

**Background:**

Community engagement has increasingly received attention in malaria research and programme interventions, particularly as countries aim for malaria elimination. Although community engagement strategies and activities are constantly developing, little is known about how those who implement research or programmes view community engagement. This article explores the perspectives of researchers and policy makers in the Greater Mekong Sub-region (GMS) on community engagement for malaria control and elimination.

**Methods:**

Semi-structured interviews were conducted among 17 policymakers and 15 senior researchers working in the field of malaria. All interviews were audio-recorded and transcribed in English. Transcribed data were analysed using deductive and inductive approaches in QSR NVivo. Themes and sub-themes were generated.

**Results:**

Researchers and policymakers emphasized the importance of community engagement in promoting participation in malaria research and interventions. Building trust with the community was seen as crucial. Respondents emphasized involving authority/leadership structures and highlighted the need for intense and participatory engagement. Geographic remoteness, social, cultural, and linguistic diversity were identified as barriers to meaningful engagement. Local staff were described as an essential ‘connect’ between researchers or policymakers and prospective participants. Sharing information with community members, using various strategies including creative and participatory methods were highlighted.

**Conclusions:**

Policymakers and researchers involved in malaria prevention and control in the GMS viewed community engagement as crucial for promoting participation in research or programmatic interventions. Given the difficulties of the ‘last mile’ to elimination, sustained investment in community engagement is needed in isolated areas of the GMS where malaria transmission continues. Involving community-based malaria workers is ever more critical to ensure the elimination efforts engage hard-to-reach populations in remote areas of GMS.

**Supplementary Information:**

The online version contains supplementary material available at 10.1186/s12936-022-04069-x.

## Background

Over recent years, community engagement has received increasing attention in health research and programme interventions [1, 2]. What constitutes community and engagement is however debated, particularly because of differences in overall aims [3]. The breadth of community engagement is encapsulated by one widely used definition: a process of collaborative work with a group of people affiliated by geographic proximity, interest or health issues, to address social and health challenges [4].

As malaria declines in areas, such as the Greater Mekong Sub-Region (GMS), countries are setting elimination as a goal. Concerted efforts are necessary to achieve these goals [5]. With diminished disease burden, funding and enthusiasm for case surveillance and treatment; paradoxically, more resources are needed to track down the remaining cases [6, 7]. Community engagement is increasingly recognized as a critical element in malaria elimination [6–8], for example, to ensure that last cases are identified and treated before they lead to focal outbreaks [8]. Recent studies on mass anti-malarial administration in Asia and Africa have shown how appropriate community engagement can promote uptake of interventions aimed at achieving elimination [5, 9].

Scholarship around strategies for community engagement related to malaria interventions has recently burgeoned [1, 3, 5, 8, 10–16]. This has taken place in the context of discussions about moving from a more top-down to bottom-up approaches [10, 17, 18]. In top-down approaches, prioritizing, planning and implementing a health programme occurs without the active involvement of the communities, whereas a more bottom-up approach combines agendas proposed by the community with the participation and engagement of community structures [5, 10]. Even with a participatory approach, meaningful engagement often remains elusive because of pre-existing power dynamics [19].

Community engagement tools or activities are evolving to reflect these changes. For instance, community meetings are more likely to involve open dialogue rather the top-down instructions delivered in the past [3]. In addition to providing feedback on the study design, meaningful participation entails co-executing the study [20, 21]. Involving community members rather than outsiders has the potential to adapt study plans and interventions to local values, culture and tradition [15, 22]. Engagement depends on the ‘human infrastructure’ of community members and researchers or implementers [23] in which social relationships that can engender trust are critical [1, 16, 20, 24]. Trust is commonly identified as a key element of successful community engagement [22] and to maintain trust, researchers/implementers and community members need to continuously invest in relationships through, for example, dialogue and participation to ensure issues raised by all stakeholder groups are addressed [1, 20, 24].

Field research has examined the aims and approaches of community engagement in the context of ongoing research and interventions for malaria elimination in the GMS [9, 11–16, 20–22]. Theses studies have highlighted the local characteristics of community engagement, along with the roles and contributions of actors in the community but less is known how it is perceived, and prioritized by policymakers, researchers and stakeholders who are central to malaria elimination policy and implementation. This article explores researchers and policymakers’ perspectives on the role and strategies of community engagement for successful elimination of malaria relevant for GMS nations.

## Methods

Details of the methods utilized have been described previously [6, 25]. The findings presented draw from interviews conducted between October 2016 and April 2017 with a total of 32 participants. These interviews formed part of a larger programme of mixed-methods research that examined the implementation of mass anti-malarial drug administration for malaria elimination across the GMS and the community engagement that accompanied it [9, 11–16, 20–22]. The overall study design was underpinned by a critical realist approach, which recognizes that although reality can never be represented with complete accuracy, it can be described to varying degrees through scientific endeavour [26].

### Study participants

All respondents were recruited based on their expertise or decision-making roles in malaria control, prevention and elimination programmes in the GMS, such as policymakers from Cambodia, Thailand, Vietnam, Myanmar, and Laos. Policy stakeholders from supranational institutions, including the World Health Organization (WHO) and international funding agencies, such as the Clinton Foundation or the Bill and Melinda Gates Foundation, were also identified.

Respondents were identified through a combination of (1) bibliography and web searches; and, (2) snowball approaches using authors’ professional networks. The appropriateness of a potential respondent in terms of offering information to address the research question was assessed based on their web-searches for malaria-related works and contributions, and were regularly discussed among the core members of the research team. To maximize the diversity of opinions, respondents endorsing different approaches to malaria elimination in the GMS were identified and approached. Respondents who had expertise and worked in various research/programmatic interventions were purposefully identified to ensure the mix of opinions. For instance, respondents included investigators conducting studies on therapeutic interventions, whereas others were conducting studies of broad preventive strategies, which included long-lasting insecticide-treated nets (LLINs).

Based on the prepared list of potential respondents, contact details were subsequently collated and potential respondents were contacted by email. Among the list of potential respondents prepared by the research team, none explicitly refused to participate but two did not respond to repeated emails or phone calls and could not be interviewed. Recruitment was continued until theoretical saturation (whereby no novel information was forthcoming from subsequent interviews) [27]. The total sample size was not pre-determined but rather, because data analysis ran in parallel with data collection, it was possible to determine this point and stop recruitment.

### Data collection and interview guide

A topic guide for the semi-structured interviews was developed, based on the initial research questions. Topics included mass drug administration (MDA), malaria elimination and community engagement. During interviews, an iterative and flexible approach was taken to elicit in-depth information, as well as to ensure that relevant topics were not neglected. At the same time, prompts were used to ensure that respondents did not stray beyond the scope of topic guide. Adhering to these major topics, and aware of the respondents from two groups (policymakers and researchers), researchers judged the saturation of data under the topics explored [28].

All interviews were conducted between October 2016 and April 2017 at different locations in Myanmar, Thailand, Laos, Cambodia, Vietnam, and USA. Whenever possible the interviews were conducted face-to-face on the study site of a pilot malaria elimination strategy project, at an international tropical-medicine conference (American Society of Tropical Medicine and Hygiene conference-2018, where global malaria researchers convene annually), or at ministerial- or governmental offices. If a face-to-face interview was not possible, alternative procedures via Skype or telephone were selected. Two respondents could not participate in face-to-face meetings because of professional commitments and thus opted to respond to questions through emails.

All interviewers were conducted by the first author [NK], who is a medical doctor with a master’s degree in public health and tropical medicine as well as training in qualitative and quantitative social science research methods. Data collection and analysis was supervised by experienced qualitative social scientists (the second [BA] and last author [CP]). All interviews ranged from 20 to 90 min in length and were conducted in English. Interviews with policymakers and funders were longer than with malaria scientists. All interviews were audio-recorded and subsequently transcribed verbatim by an independent transcriber. The interviewer checked all transcripts for accuracy.

### Data analysis

All interview transcripts were analysed thematically using qualitative data analysis software (NVivo 11; QRS International, USA). Transcripts were read repeatedly and coded line-by-line using pre-established codes (deductive approach), and codes, which emerged during the analysis process (inductive approach). Broadly, deductive codes were derived from the topic guide (Additional file 1: Appendix S1) and inductive codes were developed in response to the emerging data that could not be captured in the initial codebook. Coding was performed independently by two researchers (NK and BA). Disagreements among the researchers were resolved by discussion. For example, initial codes drawn from the interview guide included ‘community engagement’, with ‘aims’ as a sub-code. ‘Trust’ however was subsequently added as a code to ensure that this prominent issue was incorporated and given sufficient attention. Similarly, the inductive (sub) code of ‘incentives’ was added to the code of ‘challenges’, which was used to identify challenges in community engagement and more broadly in malaria elimination.

Alongside coding, the analysis process entailed reiterative interpretation and abstraction of the codes and data, which allowed the analysists to identify and explain prominent themes, their patterns and outliers. The findings presented result from a mix of interpretation (reflection) by authors based on their expertise, experience and its relevance and subsequent abstraction to respond to the research question. The themes below, which resulted from the coding, are broad categories of meaning identified across the data set and are subdivided when necessary to capture prominent sub-themes relevant to the study aim.

### Ethical approvals

Initial data collection was conducted as part of the Targeted Malaria Elimination (TME) research project [9, 29]. TME was a cross-over community, randomized, controlled trial conducted in Thai-Myanmar border, Myanmar, Cambodia, Laos, and Vietnam which entailed mass anti-malarial administration and quarterly parasitaemia surveys for a year [29]. The ethical approvals were obtained from all countries where TME-related research was conducted: Laos: Lao National Ethics Committee for Health Research (Ref. No. 013-2015/NECHR), Government of the Lao PDR and the Oxford Tropical Research Ethics Committee (1015-13); Cambodia: National Ethics Committee for Health Research Cambodia (NECHR 0042 and 0051) and the Oxford Tropical Research Ethics Committee (OXTREC; 1017-13); Vietnam: the Institute of Malariology, Parasitology and Entomology in Ho Chi Minh City (185/HDDD), the Institute of Malariology, Parasitology and Entomology in Qui Nhon and the Oxford Tropical Research Ethics Committee (1015-13); Myanmar: Ethics Review Committee of the Department of Medical Research (Ref: 74/Ethics 2014) and the Oxford Tropical Research Ethics Committee (23-15; 1015-13), the Tak Province Community Ethics Advisory Board and the village committees. Further ethical approval was obtained from Oxford Tropical Research Ethics Committee (OxTREC), and was approved on 31 January, 2017 (OxTREC ref: 5122-16). Verbal informed consent was obtained from all respondents prior to the interviews. All respondents were given an explanation about the study, the voluntary nature of their participation and its rationale. The respondents were briefed that they can drop out of the study at any time during the interviews, without providing any justification. Confidentiality and anonymity were secured for all respondents.

## Results

A total of 32 respondents participated in the study that included 17 policymakers and 15 leading malariologists. Policymakers included three females and 14 males; researchers included three females and 12 males. The balance of policymakers and researchers allowed a fair comparison between their perspectives and to generate conclusions.

The findings are presented by themes, integrating direct responses from both researchers and policymakers:

1. Aims of community engagement;
2. Strategies for community engagement;
3. Activities or tools for community engagement; and
4. Challenges for effective community engagement.

### Aims

**Fig. 1:**
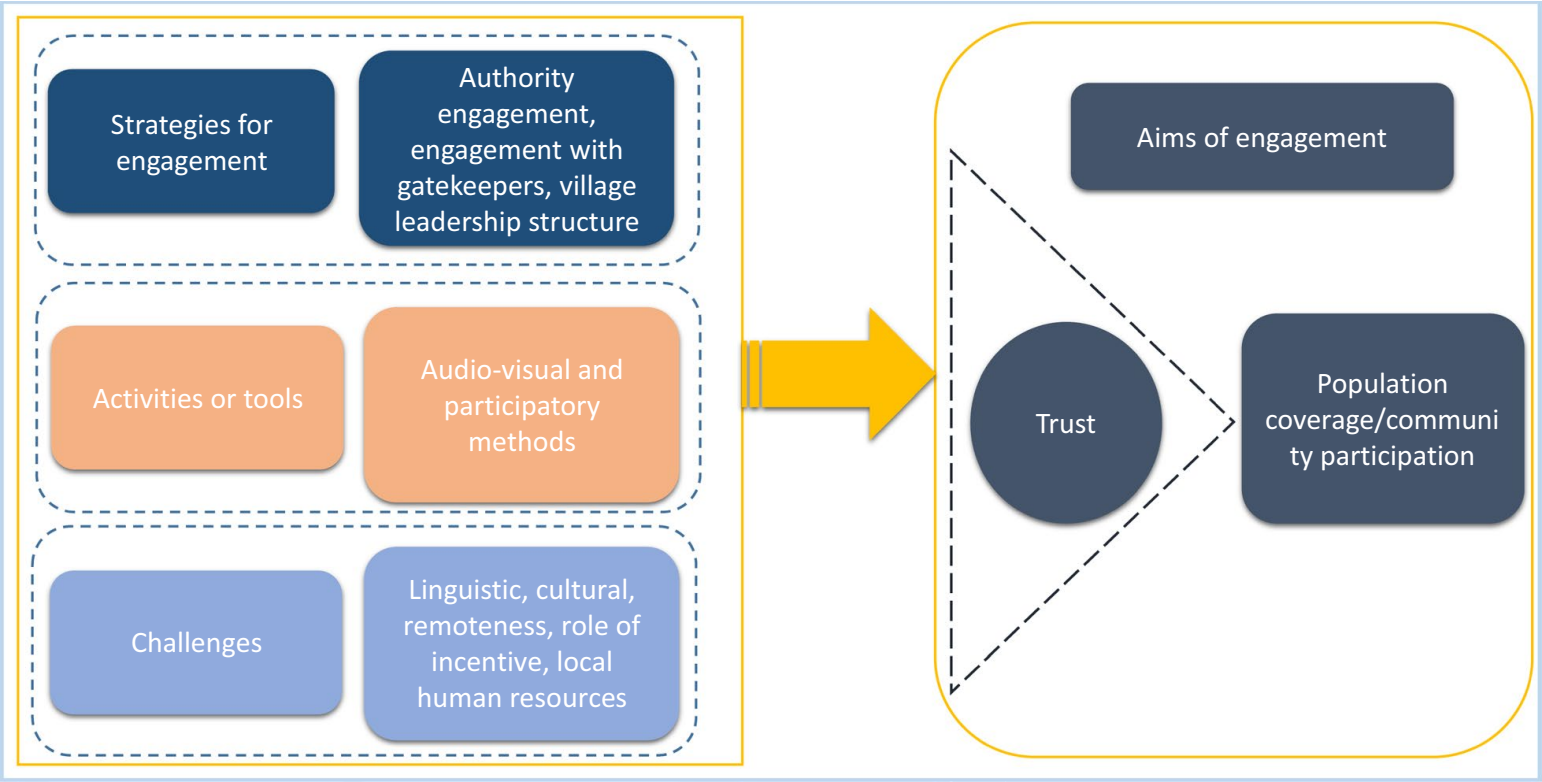
Aims, strategies and challenges of community engagement relevant for malaria elimination

Policymakers and researchers described community engagement as an important aspect of research/programmes to ensure prospective community members understand the study or programmes, their objectives, benefits and possible complications. Researchers and policymakers saw the main objectives of community engagement as promoting participation in research or programmatic interventions, and in the case of MDA, increasing population coverage (Fig. 1).

One policymaker reflected on the modelling studies related to mass anti-malarial administration in which high population coverage was emphasized as critical for the success of this intervention.

*“So, the modellers have agreed that achieving high coverage is what you need to make MDA effective. If that’s what you need to achieve, then of course that’s where your community engagement comes in, because if you don’t have a community that is convinced that this is a good idea you don’t achieve coverage, then this thing will not be effective.”* (SSI-P14).

Others reflected generically about why and how community engagement should be an integral part of the research or intervention.

*“… I think community engagement is a key thing. The community beforehand should be convinced, should be informed about the remedy and its benefit and so on and so forth …”* (SSI-P8).

At the time of the interviews, several MDA trials were ongoing in Southeast Asia and potential respondents were presented with a precis of these trials as part of the information sheet. Researchers generally expressed the outcome of community engagement in terms of MDA studies, particularly the critical role of high participation/population coverage in the success of MDA.

*“Community engagement is the essential method, it is very important tool, and it is the first activity to be implemented in the malaria elimination project before we can do a[n] MDA and MDA-related activities.”* (SSI-P24).

### Trust

Policymakers and researchers highlighted that the goal of community engagement was to build trust among community members, which could ultimately affect participation. Nevertheless, they mentioned several challenges that may have an impact on achieving this goal. With regard to selecting an appropriate person for community engagement in the community, someone who is respected and popular in community was deemed to garner more trust than a researcher who is an outsider to the community.

*“They know the governor, they know the district governor, they know the community chief. [It is] not us who make [build] the trust; we use these people to make [build] the trust.”* (SSI-P13).

Reflecting on the MDA studies, a few policymakers mentioned that when community members experienced adverse events due to the anti-malarials offered, the relationships with and trust in the researchers (and their representatives) played a critical role in maintaining the acceptance of the MDA. A few policymakers also clearly stated that early and well-planned engagement strategies is critical, in contrast to engagement that reacts to setbacks in the community. Such setbacks and failures, often due to under-resourced or poorly implemented engagement strategies, have reputational damage that can erode trust for future community engagement efforts and research.

When it came to local politics, researchers described challenges in politically divided communities, where the even slight (perceived) favouring of one group can mean opposition towards the research from another group. If this happened all trust could be destroyed.

*“If you are perceived to be sided for one group or another, or sided with the government or against, then you are dead. You have to gain the trust of everybody and that’s what we have been doing in community engagement. In fact, community engagement is only about trust. Trust at the highest level – your level one, your level two, your level three and all the way to the…… I would say in this case, the level five, which is the people is much more important; at the village level.”* (SSI-P27).

Researchers also reported the importance of historical legacies of research, and community members’ past experience with the institution and researchers. Community members may be suspicious of the true objectives of the study intervention because of negative experiences with previous projects and this may lead to difficulties in trust building and may threaten the study.

*“And we worked in a village 10 years ago …, and we had been taking blood, and there was lots of discussion where we agreed that we could do that. And then we asked to take stools as well. And the village committee met and discussed our request to have stool samples. And we were sitting in the school on the little chairs, right? And the committee came in, a file of elderly men, and they looked terribly severe and unhappy. And the village head said, "We've considered." XXXX translated. It was in ‘Lao theung’. It was in Lao. The village head said, "We've considered your request, and we agree." And they all laughed and banged their hands on the table and said, "Because we were very worried that you might sell our blood, but we're pretty damn sure you're not going to sell our stool.”* (SSI-P30).

One researcher pointed out that, to gain trust, it is crucial that the study team takes responsibility for any potential complications related to the study that may occur. According to the researcher many participants are not just worried about their own health but also of the potential consequences that complications related to the study that may bring to their families if they cannot work and support their family anymore.

### Strategies

Most policymakers agreed that the initial approach should go through the leadership of the community,

*“You engage first with the leaders and then once they have understood the story on why you are there and what is going to happen and go and inform the rest of the population…”* (SSI-P1).

Many policymakers also stressed the importance of a formal approach, for example with documentation of official approval. One policymaker favoured starting community engagement with a small group within the community who then can function as a bridge to other community members who may be more difficult to access and to convince to participate. Several policymakers pointed out that understanding the structure of the community’s leadership, if possible, through local partners, makes it a lot easier to find out on who to contact first.

*“…find out specifically, every locale who we need to talk to, who the decision makers are. You know, maybe if you want to talk to the village chief but he has actually had a 50-year multi generation l feud with some other family over here. We always go through local partners who we hope to have that answer or have that kind of local information.”* (SSI-P23).

Most researchers agreed with policymakers’ opinion to initiate a community approach through the village leaders “*and for them to have a public meeting and discuss with the community.”* (SSI-P30). Those village leaders may have different positions within the community and, as mentioned above, should be selected if possible with the help of local partners.

*“First contact was usually made by village leaders, spiritual leaders, the health workers discuss with them and then they kind of organize further steps.”* (SSI-P25).

### Activities

Policymakers and researchers detailed a variety of activities that were and could be potentially appealing to community members. Most of the respondents described audio-visual methods to communicate with community members was important. Among those mentioned were focus groups *“… bringing everyone together, bringing up the leader and the one with influence…”* as well as other activities as drama, concerts, dances and showing movies. *“If you have DVDs, the TV is super…”* (SSI-P1).

Respondents described other tools, such as the use of mass media, leaflet, handouts and visual representations. All these tools were thought to be more comprehensible for community members with poor literacy as well as attractive and entertaining. Policymakers emphasized the need to utilize the latest technologies to reach community members.

*“Mass media campaigns through radio channels, with the very famous local program to ensure that people are aware of what is going to happen. We do almost a month of this before we start to do the MDA.”* (SSI-P16).

Most researchers stressed the need to visualize messages, including the study/programme objective, rather than just brief them to community members.

*“… we showed to each other village; we showed what was happening in the other villages. We had maps and we had graphs and things that are easy to visualize. Said you see in that other village over there; we did the same program and look at the number of cases. Last month they had no case of malaria. But your village, it’s going up and up. Then people start to realize…”* (SSI-P27).

Regardless of advancement in technologies, meetings were also described as crucial, conventional and comprehensive platform to get researchers’ message get across.

*“The most effective, right? I think, from what I observed—the meeting, the meeting with the villagers”* (SSI P13)

A researcher also reflected on the MDA study in Laos, and emphasized the value of meetings where visually appealing posters were an important tool to discuss about the study.

*“The village where we worked with ethnic minority, we explained using posters, [which] people understand best.”* (SSI-P29)

Several of these tools were mentioned by researchers and policymakers and they also emphasized entertaining activities, such as community games, were essential to boost familiarity and build relationships in the community.

### Challenges

Researchers and policymakers highlighted several challenges for effective community engagement. One policymaker highlighted naivety, with regard to understanding the community, as a widespread issue that has caused problems in several interventions in the past.

*“This broad assumption that you can just walk up and go to people’s place and assume that A) they’re there, as if the whole of the community sits in a hut waiting for public health people to walk up, and B) you will just take whatever the people tell you to take is a mind-blowing assumption in my opinion. I don’t know where that comes from.”* (SSI-P14)

One challenge was that researchers pointed out the lack of universally applicable principles or strategies for community engagement. Researchers also mentioned the fact that (human and financial) resources were often insufficient for thorough community engagement. The high costs and effort was described a main reason why several governmental institutions are often hesitant in regard to implementing large-scale community engagement.

Respondents also identified challenges related to ethnic, linguistic and cultural diversity, which can require resources when conducting engagement. Selecting a suitable and applicable approach to such a heterogenous group of community members was reported to be a major challenge and referred to Laos where there are scores of ethnic groups. Other challenges raised by respondents were due to remoteness and poor accessibility to the villages where malaria is endemic.

### Incentives

The use of incentives was described as a very delicate topic in community engagement. Only a few researchers made the distinction between various forms of payment (reimbursement, compensation, incentives) made to research participants. The term ‘incentives’ was seemingly used by respondents to mean any form of material benefits offered to research participants.

The definition of what is an incentive and whether giving incentives is acceptable differed within and between the respondent groups. Most policymakers opposed incentives in terms of cash payments due to ethical concerns. Some offered examples of how incentives (including the amount) are interpreted in the study context, with some, community members interpreting incentives as payment in exchange for participation.

*“I think that is unethical. You cannot pay people to take drugs. This, I think, is forbidden by ethical committees.”* (SSI-P5)

Nonetheless, policymakers did agree that participants needed to be compensated for their loss of opportunity and incurred costs attached to participation in the research.

*“That they come and listen to you, or attend your MDA, whatever. What do they lose by not going to the rice field? These things need some compensation.”* (SSI-P6).

Researchers justified payments if deemed to be a compensation for lost opportunity or income for their participation in research but not as material benefits) to promote participation. A few respondents, mostly policymakers raised an important issue of sustainability of offering incentives when it came to rolling out large-scale interventions or programmes. In contrast, most researchers considered compensation a necessary and crucial tool in recruitment. They saw payments as an integral component of standard research recruitment where potential participants need to be compensated for lost opportunity and income.

*“Lost income for an intervention which is, at this stage, experimental. You should pay for it.”* (SSI P32).

Other researchers stressed the importance of offering a community incentive rather than individual incentives. This was particularly relevant for very large-scale studies or programmes, for which individual incentives would be a burden in terms of resources and time. A few researchers also pointed out the vulnerability of participants, especially in poor communities, where incentives may be important to ensure community members participate. Researchers also explained the lack of essential healthcare in remote communities, implying offering healthcare was thought to be important.

*“At least a few more services at that health post, not just malaria, but you have a little bit of paracetamol, penicillin, whatever.”* (SSI-P26).

### Human resources

Human resources were deemed central in community engagement by researchers and policymakers. Several policymakers reported challenges in finding culturally competent staff with proper *“education…writing and reading abilities… languages.”* (SSI-P6).

A few policymakers described the option of involving prominent figures within the community, for example local healers, who may be willing to cooperate and provide access to other community members,

*“…with the benefit of this very deep, local, ethical, logical knowledge that is very locale specific we were able to convince to refer kids with that specific diagnosis to the hospital for the research team supplementing the local doctors to treat with proper anti-malarial.”* (SSI-P23)

Several senior researchers stressed the importance of working with local staff to build the knowledge base and to implement the research/project efficiently.

*“On the knowledge of the people who are doing the work, which are most of the people in this unit, … people from the local population. 90% of the people who do the work are Karen or Burmese or Thai. If you listen to them, they tell you a lot of things. They tell you that in this place, no, you can’t go. We cannot. They know who to talk to be able to approach person X, Y and Z.”* (SSI-P27)

One of the researchers emphasized that specifically in the beginning of the intervention it is crucial to have local- and well-respected people on the team.

*“Who know how to build a team which is going to be accepted, recognized and trusted by whatever community.”* (SSI-P27)

Even local recruitment was handed over to a locally respected person to support finding staff who are also respected by the community members *‘We usually hired village leaders or some of his people [familiar person] to find people..”* (SSI-P25)*.*

## Discussion

As malaria continues to decline across the GMS, there is a potential for complacency just when greater efforts are needed to reach elimination goals [6, 7]. Countries in the GMS are embarking in the last phase, referred to as ‘last mile’, where the last pockets of malaria are found among the remote and hard-to-reach populations (e.g., mobile and migrant population, such as forest workers) mostly living around the villages near the forest, forest fringe and mountainous areas [30]. The last mile to elimination is where participation of communities is particularly important, because as cases go down, surveillance needs to be strengthened and interventions targeted. Outbreaks in elimination settings can spark further transmission and reverse gains. Furthermore, as falciparum malaria declines, the burden of vivax malaria becomes increasingly apparent [31]. The elimination of vivax malaria is complicated by residual hypnozoites that can re-activate years later [31, 32]. To eliminate malaria completely, sustained community engagement that addresses this complexity is essential.

In the context of declining malaria in the GMS, this study explored the perspectives of policymakers and researchers on the role of community engagement. Overall, the respondents viewed the goal of community engagement as promoting participation in malaria research or programmatic interventions. Respecting and building on existing leadership structures was seen as an essential first step in engagement. Interactions with community members that consisted of dialogue and being responsive to concerns, with research messages delivered using creative forms, such as visual methods, were deemed important. There was less consensus about the issue of incentives in research and as part of community engagement and there was some confusion about the different types of payments made. Researchers saw some sort of payment as necessary to compensate opportunity costs, particularly when participating in research. Meaningful engagement with community members that entailed participatory methods, such as recruiting local human resources and building relationships over a length of time to build trust, was deemed critical despite various challenges.

The role of trust in community engagement has been widely discussed, although it is generally described as an intermediary step to achieve an outcome [1, 20]. Building trust and relationships is critical for research or programme to succeed [20, 33, 34]. For instance, the relationship and institutional trust developed by the Medical Research Council (MRC) in The Gambia, Shoklo Malaria research Unit (SMRU) on the Thai-Myanmar border and Kenya Medical Research Institute (KEMRI) in Kenya have contributed to community participation in clinical or non-clinical research [3]. The interviewed policymakers and researchers, however, described building trust as a goal in itself.

Trust can take a variety of forms, for example, between individuals (interpersonal trust), or between a person and an institution (institutional trust) [35, 36]. Trust is often based on positive relationship with communities. To build such relationships, researchers have described the value of involving persons who are familiar and are trusted by potential participants. This may mean collaborating with an influential or popular leader. Yet this approach has potential pitfalls in politically divided communities [15, 22]. Once settled in a community, programme staff and researchers can build relationships and trust through their presence and interactions over time [37, 38]. These relationship are however affected by the institutions to which they are connected and past experiences of the institution, and its wider reputation [36]. Local staff can struggle to balance their positions as a representative of a research institution or implementor and a member of the community [38]. Staff based in communities also experience dilemmas when trying to abide by the research protocol but being aware of the realities and social relationships in the communities. For instance, providing assistance to a non-participant may fall outside the scope of a study protocol where a field staff may feel vulnerable in his/her desire to be of help [39]. Future research and programme implementation should invest in preparing community-based staff for such situations.

Continued support and motivations to community-based malaria workers are essential in sustaining the malaria elimination efforts [40]. With the decline in malaria burden in GMS, the roles and responsibilities of village malaria workers (VMWs) are in flux and this may adversely affect last mile malaria elimination efforts. This last mile requires engaging hard-to-reach populations, such as forest workers, mobile and migrant populations in remote regions, a task for which VMWs are critical. Efforts to sustain and expand the roles and capacities of VMWs are critical to maintain the hard-achieved gains in malaria prevention and control.

One of the first steps in initiating community engagement for research and programmatic intervention is engaging with authorities at all levels in a community’s leadership structure [22, 41]. In the shift from conventional vertical programmes to horizontal and community-directed approaches, authority engagement seems out of place [5, 10, 42]. This may have resulted in authority engagement being inconsistently articulated in research on community engagement. Respecting and engaging with authorities is critical to capitalizing on the existing social network and influence borne by the structure. Embedded within the authority and leadership structure is legitimacy and institutional trust that takes many years to develop [36]. Most research and programmatic interventions in certain ways engage with the authorities and draw on their legitimacy, trust and legacy [36]. Nonetheless, sometimes relying on formal authorities alone can have adverse consequences, particularly in politically divided communities [11, 12, 43]. Engaging authority figures was invariably reported to be a critical step but both policymakers and researchers emphasized that it cannot replace engagement with community members.

Researchers and policymakers recommended engaging community members as genuinely as possible. Nonetheless, the relationships are largely predicated on and constrained by factors beyond the control of the study team. Such challenges include budget constraints, study duration and time spent in communities, and local barriers, such as war, conflict, linguistic and ethnic diversity, political division/partisanship, infrastructure problems, and limited human resources [6, 25].

Community engagement was seen as key for effective communication with community members. In the GMS, malaria transmission is now generally found in isolated communities along international borders where literacy levels are often particularly low. This presents particular challenges for researchers and programme staff who must ensure that community members understand the research/intervention [44]. Explaining the research message in creative ways, such as utilizing participatory drama [45], art and theatre [46], pictorial/visual description of the study [22], and audio-visual methods can assist communicate information about the study or intervention and improve participation.

Reimbursement, compensation and incentives have different meanings, implications and impact in research and health programmes [47]. Reimbursement (in cash or other forms) is intended to cover costs incurred during participation in the research or programme. Compensation refers to paying for opportunity costs, and incentives can be both material and non-material rewards for participation [47]. Policymakers echoed concerns about payments in general, particularly that they could be unsustainable and potentially trigger “undue inducement” to participate in research/programmes [48]. Researchers saw reimbursement and compensation as an essential feature of participation, and deemed it unethical not to provide reimbursement and compensation.

Differences of opinion between policymakers and researchers related to providing payments to potential participants were likely rooted to their experience and the practices of research and programmes: community members are far less likely to receive any payments for participating in a programmatic intervention compared to research. Researchers saw providing health services where needed (ancillary care) and community benefits rather than individual benefits as more appropriate in many remote and underprivileged region where malaria research is undertaken [9, 12]. In the broader global health scholarships, offering benefits, such as health services or other sorts of payments, to participants in less-privileged communities reflects existing power and wealth inequalities [48–54]. In that sense, researchers and research institutions could offer sustainable solutions to these embedded structural differences, which, for example can include promoting local employment, skills and capacity development, and contribution in promoting health services.

Human resources are pivotal to community engagement because researchers who interact with the community are seen to be representative of the research or programme [22]. Community members place their trust in staff who they interact with based on who they are (competence), institutional affiliation (institutional trust) and their local cultural knowledge [36]. Research representatives, preferably community members with shared cultural knowledge are preferred over outsiders to facilitate communication [55]. Also, a community member will always be accessible regardless of the scope and duration of the research project. This also means that the community member who is a resident can maintain a presence in the community, which can strengthen accountability, responsiveness and a sense of support to the community members.

Despite these advantages, community members also evaluate the representatives in terms of who the person is, their demeanour, political affiliation, interests, reputation, and competence. Thus, it is essential that local human resources are congenial to all community members. Employing community members who are selected by the research team also means that they are seen to be part of the research team rather than a community member [37]. Tensions and jealousies could also arise because of the selection process perceived as unfair [56, 57]. A transparent recruitment process led by the community can help to overcome some of these challenges.

## Strengths and limitations

Respondents included a diverse group of policymakers and researchers based in Ministries of Health across the GMS and in research institutions located in several GMS states. Interviews were conducted both online and in-person. Many of the respondents had full agendas and the flexibility of online interviewing meant that a larger and more diverse group could be recruited. Conducting the interviews in English was one potential limitation although all but one of the respondents was comfortable with this language and both policymakers and researchers were accustomed to participating in international events for which the *lingua franca* was English. One interview responded to written interview questions by email. Although the responses were likely influenced by the different format, this enabled the inclusion of the perspective of a policymaker who was less comfortable with English.

## Conclusion

In the context of declining malaria transmission and aims to reach elimination in the GMS states, researchers and policymakers in the region emphasized the importance of community engagement in promoting participation in research and intervention programmes. They saw building trust with the community as an important way to achieve this. Involving community authority/leadership structures was seen as key to engagement activities, although they recognized that this required attention to political affiliations and other social relationships. They identified constraints, such as geographic remoteness, and social, cultural and linguistic diversity as barriers to meaningful engagement. Local staff were viewed as critical in community engagement who could connect researchers or policy makers with prospective participants. Sharing information with community members, using various tools, for example, creative and participatory messages, was highlighted. Given the difficulties of the last mile to elimination, sustained investment in community engagement is needed in isolated areas of the GMS where malaria transmission continues. Particularly, as malaria continues to decline in the region and recedes to remote and hard-to-reach areas, it is essential to engage community members, stakeholders and specifically village malaria workers or community health workers who can reach out to the affected population and sustain elimination efforts.

## Supplementary Information

**Additional file 1.** Topic guide.

## Data Availability

The data is available upon request to the Mahidol Oxford Tropical Medicine Research Unit Data Access Committee (http://www.tropmedres.ac/data-sharing) complying with the data access policy (http://www.tropmedres.ac/_asset/file/data-sharing-policy-v1-0.pdf).

## Abbreviations

CE: Community engagement
GMS: Greater Mekong Sub-region
MDA: Mass drug administration
ME: Mass elimination
TME: Targeted Malaria Elimination
WHO: World Health Organization
