# Supplementary material for: Community engagement for malaria elimination in the Greater Mekong Sub-region: a qualitative study among malaria researchers and policymakers
Source: Malar J. 2022 Feb 14;21:46. doi: 10.1186/s12936-022-04069-x (PMC8845385; doi:10.1186/s12936-022-04069-x)
Supplement: Supplementary file 1 — Additional file 1. Topic guide. [file 12936_2022_4069_MOESM1_ESM.docx]

**Appendix 1: Topic Guide**

***Questions will be open and should only be guided by the following questions***

**Level I:**

**Earlier involvement and knowledge on Malaria-Elimination (ME)**

1. How were you involved in malaria-elimination/prevention/control program? When? Where? Why?
2. What do you think about ME-feasibility, practical issues, rigorosity of science in ME program, ethics, and outcome?
3. What do you think are important tools in ME – with focus on the SEA region?
4. What were the challenges (opposition, funding, lack of evidence, lack of confidence)?

**MDA as a tool for Malaria Elimination**

1. Are you familiar with the use of MDA in ME?
2. What are your thoughts on that?
3. Have you been involved in MDA projects before?
   - If yes, where, when and what was your role/function?
4. Based on your experiences, how is your view on MDA as a tool for ME?
5. What are the benefits of using MDA?
6. Is there evidence lacking in using MDA? Is more evidence needed (different regions, wider areas, different populations, etc.?)
7. Is there enough evidence in favor of MDA to implement it as an important tool in ME?
8. Would you recommend MDA as an important part of ME? On a global level?

**Practical implementation of MDA and challenges**

1. How could MDA be scaled up, could MDA be implemented in different, greater regions?
2. What is your opinion on cost-effectiveness on MDA?
   - Is it feasible from a budgeted point of view?
   - Are there other methods in ME that may be more effective-/cost effective?
3. What do you think are the most promising drugs for MDA in ME?
4. What are your ethical thoughts on MDA?
5. Challenges/Possible strategies to cope with during the implementation of project:
   - Political-, religious-, ethical-, social-issues
   - Communication-
   - Regional issues (isolation/remote areas, seasonal challenges)
   - Local opposition
   - MDA related-/unrelated health issues of participants as well as possible drug side-effects
   - Problems with staffs and logistics

**Maximizing coverage and CE**

1. What are the essential strategies/methods to achieve/maintain high coverage?
2. Are you familiar with the term CE?
   - If yes where and when have you heard of CE?
   - What are your experiences with CE?
3. Do you think that CE should be an important tool in future MDA/ME projects?
4. Do you think the type of CE has to be locally assessed?

**Initial outcome of TME, future directions**

1. What are your (initial) thoughts on the outcome of ME?
2. Indicators for a rather successful or unsuccessful intervention?
3. What do you think will be the next stage of ME?

**Level II:**

**Earlier involvement and knowledge on Malaria-Elimination**

1. How were you involved in malaria-elimination/prevention/control program? When? Where? Why?
2. How was ME introduced to you?
3. What do you think about ME?
4. What do you think are important tools in ME – with focus on the SEA region?
5. Have you been personally involved in ME implementation?
   - If yes, where, when and what was your role/function?
6. What were the challenges (opposition, funding, lack of evidence, and lack of confidence?

**MDA as a tool for Malaria Elimination**

1. Are you familiar with the use of MDA in ME?
2. What are your thoughts on that?
3. Have you been involved in MDA projects before?
   - If yes, where, when and what was your role/function?
4. Based on your experiences, how is your view on MDA as a tool for ME?
5. What are the benefits of using MDA?
6. Is there evidence lacking in using MDA? Is more evidence needed (different regions, wider areas, different populations, etc.?)
7. Is there enough evidence in favor of MDA to implement it as an important tool in ME?
8. Would you recommend MDA as an important part of ME? On a global level?

**Practical implementation of MDA and challenges**

1. How have you actually (would you) implemented MDA in the field?
   - Implementation through political instances (top-down approach)
   - Involving local staff: local health-staff (for example. malaria workers), local political and/or religious-/spiritual leaders, local persons of respect, business leaders.
   - Involving external help: university students, paid workers?
   - How would you involve-/motivate locals to get and stay involved?
   - Give incentives? Social-/educational- events? Keeping close contact/relation with the participants? Other activities of CE?
   - How to monitor MDA outcome, follow up on MDA?
   - How did/would you prevent local opposition? How would/did you deal with opposition?
   - How did/would you choose the location (For example: based on parasitemia, guided by site researchers, government, other stake holders)?
2. How could MDA be scaled up, could MDA be implemented in different, bigger regions?
3. What is your opinion on cost-effectiveness on MDA?
   - Is it feasible from a budgeted point of view?
   - Are there other methods in ME that may be more effective-/cost effective?
4. What do you think are the most promising drugs for MDA in ME?
5. What are your ethical thoughts on MDA?
6. Challenges/Possible strategies to cope with during the project:
   - Political-, religious-, ethical-, social-issues
   - Communication (communication difficulties-modesty can cloud actual opinion of persons, information lag, information gaps)
   - Regional issues (isolation/remote areas, seasonal challenges)
   - Local opposition
   - MDA related-/unrelated health issues of participants as well as possible drug side-effects
   - Problems with staffs and logistics

**Maximizing coverage and CE**

1. What do you think would be effective to get/maintain high coverage?
2. Are you familiar with the term CE?
   - If yes where and when have you heard of CE?
   - What are your experiences with CE?
3. Do you think that CE should be an important tool in future MDA/ME projects?
4. Do you think the type of CE has to be locally assessed?

**Initial outcome of TME, future directions**

1. What are your (initial) thoughts on the outcome of ME?
2. Indicators for a rather successful or unsuccessful intervention?
3. What do you think will be the next stage of ME?

**Level III**

*(Possible addition to already collected data)*

**Earlier involvement and knowledge on Malaria-Elimination**

1. How were you involved in malaria-elimination/prevention/control program? When? Where? Why?
2. How was ME (Malaria Elimination program) introduced to you?
3. What do you think about ME-feasibility, practical issues, rigorosity of science in ME program, ethics, and outcome?
4. What do you think are important tools in ME – with focus on the SEA region?
5. What were the challenges (opposition, funding, lack of evidence, lack of confidence)?

**MDA as a tool for Malaria Elimination**

1. Were you familiar with the use of MDA in ME?
2. What were your initial thoughts on that?
3. Have you been involved in MDA projects before?
   - If yes, where, when and what was your role/function?
4. Based on your experiences, how is your view on MDA as a tool for ME?
5. What are the benefits of using MDA?
6. Is there evidence lacking in using MDA? Is more evidence needed (different regions, wider areas, different populations, etc.?)
7. Is there enough evidence in favor of MDA to implement it as an important tool in ME?
8. Would you recommend MDA as an important part of ME? On a global level?

**Practical implementation of MDA and challenges**

1. How have you actually implemented MDA in the field?
   - Implementation through political instances (top-down approach)
   - Involving local staff: local health-staff (fx. malaria workers), local political and/or religious-/spiritual leaders, local persons of respect, business leaders.
   - Involving external help: university students, paid workers?
   - How would you involve-/motivate locals to get and stay involved?
   - Give incentives? Social-/educational- events? Keeping close contact/relation with the participants? Other activities of CE?
   - How would you give information about MDA?
   - How to monitor MDA outcome, follow up on MDA?
   - How would you prevent local opposition? How to deal with opposition?
   - How would you choose the location (based on parasitemia)?
2. How could MDA be scaled up, could MDA be implemented in different, bigger regions?
3. What is your opinion on cost-effectiveness on MDA?
   - Is it feasible from a budgeted point of view?
   - Are there other methods in ME that may be more effective-/cost effective?
4. What do you think are the most promising drugs for MDA in ME?
5. What are your ethical thoughts on MDA?
6. Challenges/Possible strategies to cope with during the project:
   - Political-, religious-, ethical-, social-issues
   - Communication-
   - Regional issues (isolation/remote areas, seasonal challenges)
   - Local opposition
   - Have there been issues with participants?
   - MDA related-/unrelated health issues of participants possible drug side-effects
   - Problems with staff
   - Have finances been a problem?
   - Has TME been welcome to the communities?
   - Was there good local support?
   - Was TME well understood by both the staff and the participants?
   - Have there been issues with staff (incl. health issues)?
   - Have there been logistical problems?
   - Has the protocol been changed during the study?
   - Have problems been detected early? How and by whom?
   - Have there been immediate interventions when detecting problems?
   - Have there been regular briefings, meetings to discuss possible problems?
   - Did the participants have good opportunities to give feedback during and after the study?

**Maximizing coverage and CE**

1. What do you think would be effective to get/maintain high coverage?
2. Are you familiar with the term CE?
   - If yes where and when have you heard of CE?
   - What are your experiences with CE?
3. Do you think that CE should be an important tool in future MDA/ME projects?
4. Do you think the type of CE has to be locally assessed?

**Initial outcome of TME, future directions**

1. What are your (initial) thoughts on the outcome of ME?
2. Indicators for a rather successful or unsuccessful intervention?
3. What do you think will be the next stage of ME?

**Level IV**

*(Possible addition to already collected data)*

**Earlier involvement and knowledge on Malaria-Elimination**

1. How were you involved in malaria-elimination/prevention/control program? When? Where? Why?
2. How was ME (Malaria Elimination program) introduced to you?
3. What do you think about ME-feasibility, practical issues, rigorosity of science in ME program, ethics, and outcome?
4. What do you think are important tools in ME – with focus on the SEA region?
5. What were the challenges (opposition, funding, lack of evidence, lack of confidence)?

**MDA as a tool for Malaria Elimination**

1. Were you familiar with the use of MDA in ME?
2. What were your initial thoughts on that?
3. Have you been involved in MDA projects before?
   - If yes, where, when and what was your role/function?
4. Based on your experiences, how is your view on MDA as a tool for ME?
5. What are the benefits of using MDA?
6. Is there evidence lacking in using MDA? Is more evidence needed (different regions, wider areas, different populations, etc.?)
7. Is there enough evidence in favor of MDA to implement it as an important tool in ME?
8. Would you recommend MDA as an important part of ME? On a global level?

**Practical implementation of MDA and challenges**

1. How have you actually implemented MDA in the field?
   - Implementation through political instances (top-down approach)
   - Involving local staff: local health-staff (fx. malaria workers), local political and/or religious-/spiritual leaders, and local persons of respect, business leaders.
   - Involving external help: university students, paid workers?
   - How would you involve-/motivate locals to get and stay involved?
   - Give incentives? Social-/educational- events? Keeping close contact/relation with the participants? Other activities of CE?
   - How would you give information about MDA?
   - How to monitor MDA outcome, follow up on MDA?
   - How would you prevent local opposition? How to deal with opposition?
   - How would you choose the location (based on parasitemia)?
2. How could MDA be scaled up, could MDA be implemented in different, bigger regions?
3. What is your opinion on cost-effectiveness on MDA?
   - Is it feasible from a budgeted point of view?
   - Are there other methods in ME that may be more effective-/cost effective?
4. What do you think are the most promising drugs for MDA in ME?
5. What are your ethical thoughts on MDA?
6. Challenges/Possible strategies to cope with during the project:
   - Political-, religious-, ethical-, social-issues
   - Communication-
   - Regional issues (isolation/remote areas, seasonal challenges)
   - Local opposition
   - Have there been issues with participants?
   - MDA related-/unrelated health issues of participants possible drug side-effects
   - Problems with staff
   - Have finances been a problem?
   - Has TME been welcome to the communities?
   - Was there good local support?
   - Was TME well understood by both the staff and the participants?
   - Have there been issues with staff (incl. health issues)?
   - Have there been logistical problems?
   - Has the protocol been changed during the study?
   - Have problems been detected early? How and by whom?
   - Have there been immediate interventions when detecting problems?
   - Have there been regular briefings, meetings to discuss possible problems?
   - Did the participants have good opportunities to give feedback during and after the study?

**Maximizing coverage and CE**

1. What do you think would be effective to get/maintain high coverage?
2. Are you familiar with the term CE?
   - If yes where and when have you heard of CE?
   - What are your experiences with CE?
3. Do you think that CE should be an important tool in future MDA/ME projects?

**Initial outcome of TME, future directions**

1. What are your (initial) thoughts on the outcome of ME?
2. Indicators for a rather successful or unsuccessful intervention?
3. What do you think will be the next stage of ME?

**Level V**

*(Possible addition to already collected data)*

**Earlier involvement and knowledge on Malaria-Elimination**

1. How were you involved in malaria-elimination/prevention/control program? When? Where? Why?
2. How was ME introduced to you?
3. What do you think about ME?
4. Do you think it was difficult to perform ME in your region (not understanding the project, opposition, etc.)?

**MDA as a tool for Malaria Elimination**

1. Did you understand the use of drugs during the project?
2. What were your initial thoughts on that?
3. Have you been involved in MDA projects before?
   - If yes, where, when?

**Practical implementation of MDA and challenges**

1. How was MDA implemented in your region?
   - Implementation through political instances (top-down approach)
   - Involving local staff: local health-staff (fx. malaria workers), local political and/or religious-/spiritual leaders, and local persons of respect, business leaders.
   - Involving external help: university students, paid workers?
   - How were participants motivated to participate: Given incentives? Social-/educational- events? Keeping close contact/relation with the participants? Other activities of CE?
   - How could you better involve-/motivate locals to participate and to stay involved?
   - How would you improve giving information on ME/MDA?
   - How to monitor MDA outcome, follow up on MDA?
   - How would you prevent local opposition? How to deal with opposition?
2. Challenges/Possible strategies to cope with during the project:
   - Political-, religious-, ethical-, social-issues
   - Communication-
   - Regional issues (isolation/remote areas, seasonal challenges)
   - Local opposition
   - Have there been issues with participants?
   - MDA related-/unrelated health issues of participants possible drug side-effects
   - Has TME been welcome to the communities?
   - Was there good local support?
   - Was TME well understood by both the staff and the participants?
   - Have there been conflicts with staff?
   - Have problems been detected early? How and by whom?
   - Have there been immediate interventions when detecting problems?
   - Have there been regular briefings, meetings to discuss possible problems?
   - Did you have good opportunities to give feedback during and after the study?

**Maximizing coverage and CE**

1. Are you familiar with the term CE?
   - If yes where and when have you heard of CE?
   - What are your experiences with CE?
   - Do you think CE was well received by the community?
2. What do you think would be more effective to increase coverage?
3. Do you think that CE should be an important tool in future MDA/ME projects?

**Initial outcome of TME, future directions**

1. What are your (initial) thoughts on the outcome of ME?
2. Indicators for a rather successful or unsuccessful intervention?
3. Do you think you region/village has profited of the project?
